# Diffusion MRI Tractography of the Developing Human Fetal Heart

**DOI:** 10.1371/journal.pone.0072795

**Published:** 2013-08-26

**Authors:** Choukri Mekkaoui, Prashob Porayette, Marcel P. Jackowski, William J. Kostis, Guangping Dai, Stephen Sanders, David E. Sosnovik

**Affiliations:** 1 Martinos Center for Biomedical Imaging, Department of Radiology, Massachusetts General Hospital and Harvard Medical School, Boston, Massachusetts, United States of America; 2 Pathology Registry, Departments of Cardiology, Pathology and Cardiac Surgery, Boston Children’s Hospital, Harvard Medical School, Boston, Massachusetts, United States of America; 3 Department of Computer Science, Institute of Mathematics and Statistics, University of São Paulo, São Paulo, Brazil; 4 Cardiovascular Research Center, Cardiology Division, Massachusetts General Hospital and Harvard Medical School, Boston, Massachusetts, United States of America; University Hospital of Würzburg, Germany

## Abstract

**Objective:**

Human myocardium has a complex and anisotropic 3D fiber pattern. It remains unknown, however, when in fetal life this anisotropic pattern develops and whether the human heart is structurally fully mature at birth. We aimed here to use diffusion tensor MRI (DTI) tractography to characterize the evolution of fiber architecture in the developing human fetal heart.

**Methods:**

Human fetal hearts (n = 5) between 10–19 weeks of gestation were studied. The heart from a 6-day old neonate and an adult human heart served as controls. The degree of myocardial anisotropy was measured by calculating the fractional anisotropy (FA) index. In addition, fiber tracts were created by numerically integrating the primary eigenvector field in the heart into coherent streamlines.

**Results:**

At 10–14 weeks the fetal hearts were highly isotropic and few tracts could be resolved. Between 14–19 weeks the anisotropy seen in the adult heart began to develop. Coherent fiber tracts were well resolved by 19 weeks. The 19-week myocardium, however, remained weakly anisotropic with a low FA and no discernable sheet structure.

**Conclusions:**

The human fetal heart remains highly isotropic until 14–19 weeks, at which time cardiomyocytes self-align into coherent tracts. This process lags 2–3 months behind the onset of cardiac contraction, which may be a prerequisite for cardiomyocyte maturation and alignment. No evidence of a connective tissue scaffold guiding this process could be identified by DTI. Maturation of the heart’s sheet structure occurs late in gestation and evolves further after birth.

## Introduction

The myocardium is an anisotropic tissue in which the constituent cardiomyocytes (CMs) are arranged in a complex 3D network [1]. Groups of CMs in this network form laminar sheets, which allow the myocardium to shear and thicken during systole [2]. While the myocardium is a continuum, it is instructive to group aligned CMs into myofiber tracts. This approach has been used by anatomists, histologists, physicists and cardiologists to study myocardial microstructure in both health and disease [3]–[10].

In the adult myocardium, the alignment of the myocytes is highly anisotropic, creating a transmural gradient in fiber orientation [1], [7]. Fibers in the subendocardium have a positive helix angle (HA) and those in the subepicardium a negative HA, while fibers in the midmyocardium are circumferential [1], [7]. It remains unknown, however, when in fetal life and by what mechanisms this architectural pattern develops. The aim of the current study was, therefore, to use diffusion tensor magnetic resonance imaging (DTI) tractography to characterize the development of myofiber architecture in the human fetal heart. We hypothesize that this knowledge has the potential to elucidate the pathogenesis of a broad range of congenital heart defects and also to provide a valuable template for the regeneration of functional myocardium in the adult.

## Materials and Methods

Ethics Statement: The Cardiac Registry collection at Boston Children’s Hospital was started in the 1940s and includes a large number of postmortem hearts, including fetal hearts, for teaching and research. Written consent has been obtained from the parents/guardians at the time of donation to the Registry (according to the hospital policy in force at the time) stating that the hearts can be used for research purposes. However, prior to the last two decades hearts were sometimes donated anonymously to the registry for teaching and research with no personal information or identifiers. All the hearts in this study were completely anonymized prior to imaging. A research protocol to perform this study was specifically approved by the Institutional Review Board of the Massachusetts General Hospital (MGH). The hearts were imaged at the MGH in a completely anonymized fashion. Only the gestational age of the hearts was known.

Six normal human hearts were obtained from the cardiac registry collection at Children’s Hospital in Boston. The gestational ages of the five fetal hearts studied were: 10, 13, 14, 14 and 19 weeks. The sixth heart, a normal neonatal heart, was obtained 6 days post birth (P6). All hearts were fixed in formaldehyde and were structurally intact. DTI of the fetal hearts was performed at 9.4 Tesla (Biospec, Bruker, Billerica MA) with a 3D fat-suppressed single-shot spin echo EPI (echoplanar) sequence with the following parameters: spatial resolution 130×130×130 µm^3^, TR = 1000 ms, TE = 18 ms, b-value of 1400–1600 s/mm^2^, 24 diffusion-encoding directions plus one b = 0 s/mm^2^ image. DTI of the P6 (day 6 neonatal heart) was performed using the identical sequence at 4.7 Tesla (Biospec, Bruker) with the following parameters: spatial resolution 500×500×500 µm^3^, TR = 1200 ms, TE = 41 ms, b-value of 1560 s/mm^2^, 24 diffusion-encoding directions plus one b = 0 s/mm^2^ image.

The fetal and P6 hearts were compared with each other and an adult human heart, which was imaged as previously described [10]. In brief, a 2D fat-suppressed single-shot spin echo EPI sequence was used on a 3 Tesla clinical imaging system (TRIO, Siemens, Erlangen Germany). Parameters included: voxel size = 2×2×2 mm^3^, TR/TE = 8430/96 ms, 50–70 short axis slices (without gaps) to cover the entire heart, b-value of 2000 s/mm^2^, 6 diffusion-encoding directions plus one b = 0 s/mm^2^ image, 24 averages.

In all hearts, diagonalization of the diffusion tensor in each voxel was performed to derive the primary, secondary and tertiary eigenvectors (ê_1_, ê_2_, and ê_3_) and their associated eigenvalues (λ_1_, λ_2_, λ_3_). The image acquisition parameters above were chosen to ensure that the transmural thickness of the myocardium in each heart would contain more than 8 voxels. The structural maturation of the fetal myocardium was evaluated by measuring its mean diffusivity (MD) and the degree of tissue anisotropy, estimated by the fractional anisotropy (FA) index [11], [12]. Further characterization of the diffusion tensor field within the myocardium was performed using an ellipsoid-based representation. In addition, the primary eigenvector (ê_1_) field in the lateral wall of the left ventricle (LV) was numerically integrated into streamlines using a 4^th^ order Runge-Kutta approach to create myofiber tracts [10].

Following MR image acquisition, the fetal hearts were sectioned in a plane tangential to the epicardium in the lateral wall. Phase-contrast microscopy was performed on H&E stained histological sections of the hearts at 20×magnification. Values in the manuscript are reported as the mean ± standard deviation of all voxels in a given heart. Comparison of the MD and FA values in the fetal, P6 and adult hearts was performed with ANOVA and a Tukey’s test.

## Results

**Figure 1:**
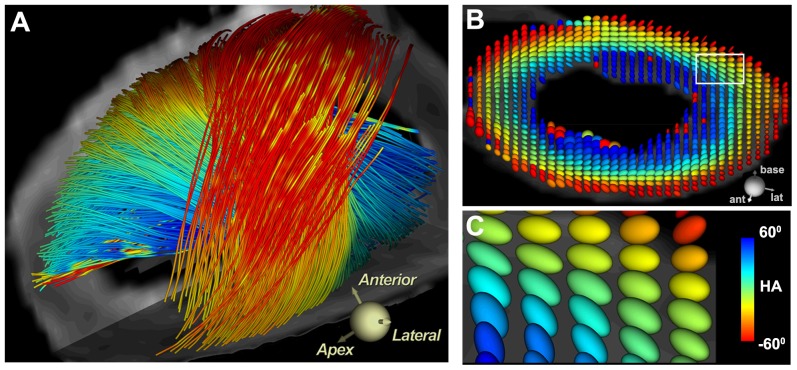
Fiber architecture in the adult human heart. (A) Fiber tracts in the lateral LV wall. The tracts are color-coded by their inclination or helix angle (HA). Tracts in the subendocardium have a positive (right-handed) HA, tracts in the subepicardium have a negative (left-handed) HA, and those in the midmyocardium are circumferential. (B) The diffusion tensor in each voxel in the LV is represented by an ellipsoidal glyph. The color and orientation of the glyph is determined by its HA (inclination angle of primary eigenvector). The shape of the glyph is determined by the relative magnitudes of the diffusion eigenvalues. In isotropic tissues the eigenvalues are similar and the diffusion glyphs are thus spherical. In anisotropic tissues the primary eigenvalue is significantly larger, producing high FA values and elliptical glyphs. (B, C) The diffusion glyphs in the adult LV have a highly ordered arrangement, show a consistent gradient in their transmural orientation and are highly elliptical. (White box in panel B = magnified area shown in panel C).

Fiber architecture in the adult LV is shown in Figure 1. The subendocardial and subepicardial fibers cross over each other at an angle of approximately 120^o^. The ellipsoidal glyphs form a coherent and ordered pattern throughout the LV and have a prolate shape representing a high degree of structural anisotropy.

**Figure 2:**
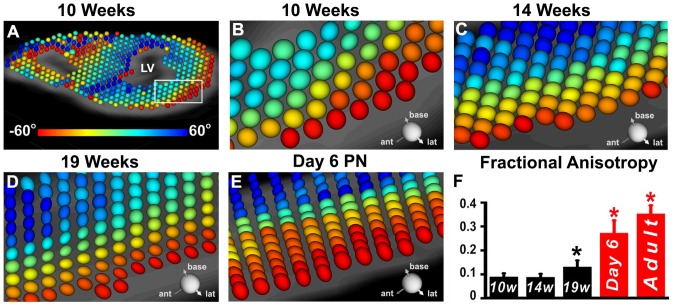
Transition from tissue isotropy to anisotropy in the developing human fetal heart. (A, B) At 10 weeks the diffusion glyphs are disordered and spherical. (B = magnified view of white box shown in panel A). (C) At 14 weeks the orientation of the glyphs resembles that seen in the adult heart but they remain highly spherical. (D) At 19 weeks of gestation the glyphs are highly ordered and are now somewhat elliptical in shape. (E) At P6 (day 6 post-natal) the orientation and elliptical shape of the glyphs is very similar to that seen in adult hearts. (F) Fractional anisotropy (FA) at 10, 14 and 19 weeks of gestation remains very low. While the coherence and orientation of the myofibers has evolved by 19 weeks, their sheet structure has not. *p<0.01.

DTI of the human fetal hearts showed that myocardial anisotropy develops fairly late in fetal life. At 10 weeks of gestation, the ellipsoids in the myocardium were spherical (all eigenvalues similar in magnitude), consistent with a largely isotropic tissue (Figure 2A–B). At 14 weeks, the ellipsoids became oblate and oriented in a transmural pattern resembling those seen in the adult heart (Figure 2C). By 19 weeks of gestation, the ellipsoidal glyphs began to assume a more prolate shape as anisotropy in the myocardium began to increase (Figure 2D).

FA in the hearts was first computed within a region-of-interest in the lateral wall and then in the entire left ventricle. FA (mean ± SD) in the lateral wall at 10 weeks of gestation was 0.09±0.01, remained at 0.09±0.01 at 14 weeks, and increased to 0.13±0.03 (p<0.01) at 19 weeks. By P6, FA in the lateral wall had increased to 0.27±0.05 (p<0.01). A corresponding decrease in MD from 1.34±0.09 at 19 weeks to 0.9±0.09 (p<0.01) at P6 was seen. The transition from isotropy to anisotropy thus started between 14–19 weeks of gestation but was a slow process, and was still incomplete at birth. FA in the lateral wall remained significantly lower in the P6 heart (0.27±0.05) than in the adult heart (0.35±0.04, p<0.01).

FA values derived from the entire left ventricle are summarized in Table 1. A steady increase was seen in FA from 14 weeks of gestation (0.07±0.02) to 19 weeks (0.1±0.04), P6 (0.2±0.08) and adulthood (0.31±0.06). Individual eigenvalues and their ratios are listed in Table 1. Little change was seen in λ_2_ and λ_3_ between 10–19 weeks. However, a 46% decrease in λ_2_ and a 51% decrease in λ_3_ were seen between the 19-week and adult hearts. The ratios of λ_1_/λ_2_ and λ_1_/λ_3_ remained low at 19 weeks (1.12±0.07 and 1.21±0.09, respectively) indicating that myocardial sheet structure had not yet matured. These ratios were significantly higher in the adult heart (1.53±0.17 and 1.83±0.23, respectively, p<0.01). A similar pattern of structural development was thus observed in the lateral wall and the entire left ventricle. Of note, significant differences in FA, the eigenvalues and their ratios were seen between the P6 and adult heart (Table 1). The λ_1_/λ_2_ and λ_1_/λ_3_ ratios in the P6 heart (1.23±0.13 and 1.53±0.5, respectively) were significantly lower than the adult heart (p<0.01), indicating that myocardial sheet architecture is not fully mature at birth.

**Table 1:** Fractional anisotropy and eigenvalues in the entire left ventricle (mean ± SD).

	Week 10	Week 14	Week 19	P6	Adult
FA	0.07±0.02	0.07±0.02	0.1±0.04	0.20±0.08	0.31±0.06
λ1	1.32±0.07	1.39±0.08	1.38±0.09	0.91±0.15	1.00±0.12
λ2	1.21±0.07	1.29±0.08	1.24±0.11	0.75±0.17	0.67±0.13
λ3	1.15±0.07	1.21±0.08	1.15±0.11	0.62±0.18	0.56±0.12
λ1/λ2	1.08±0.04	1.07±0.04	1.12±0.07	1.23±0.13	1.53±0.17
λ2/λ3	1.06±0.03	1.07±0.04	1.08±0.05	1.24±0.35	1.21±0.12
λ1/λ3	1.15±0.05	1.15±0.05	1.21±0.09	1.53±0.51	1.83±0.23

**Figure 3:**
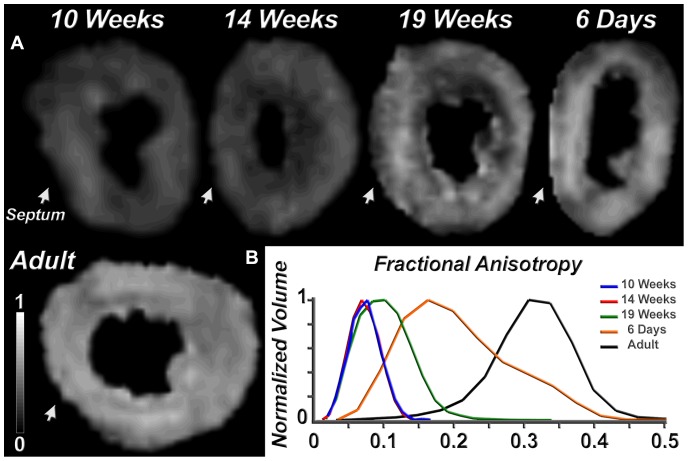
Fractional anisotropy (FA) in the developing heart. (A) FA maps of the left ventricle in its short axis are shown of a 10-week, 14-week, 19-week, P6 and adult heart. The arrowhead points to the interventricular septum. (B) Histograms of FA values in each voxel of the entire left ventricle of these hearts. The histograms at 10 and 14 weeks are virtually identical, and a shift towards higher FA values is seen only at 19 weeks. The P6 histogram contains FA values that overlap with both those seen in adult and fetal hearts. This reflects the relative structural immaturity and plasticity of the neonatal heart.

**Figure 4:**
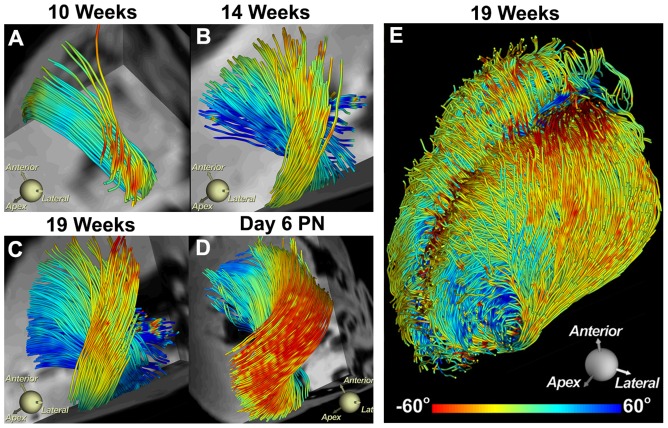
Tractography of fiber tracts (color-coded by HA) passing through a region-of-interest in the lateral LV wall. (A) At 10 weeks few tracts are present. (B) By 14 weeks tract density has increased and the crossing-helical pattern of the subendocardial and subepicardial fibers can be seen. (C) At 19 weeks the fiber tracts resemble the pattern seen after birth (D) and in adults. (PN = P6 neonatal). (E) Tractography of the entire heart at 19 weeks.

FA maps and histograms of a 10-week, 14-week, 19-week, P6 and adult heart are shown in Figure 3. The maps and histograms confirm that myofiber organization begins to develop between 14–19 weeks of gestation and continues to evolve after birth. Myofiber tractography in the lateral LV wall revealed a similar pattern of development (Figure 4). At 10 weeks of gestation few fiber tracts could be reconstructed, consistent with tissue isotropy (Figure 4A). At 14 weeks more tracts could be reconstructed but their length and density remained well below those observed in adult myocardium (Figure 4B). By 19 weeks of gestation the characteristic crossing helical pattern seen in adult hearts could be well resolved (Figure 4C, 4E). The 19-week myocardium, however, remained a weakly anisotropic tissue. While sufficient anisotropy had developed to form coherent tracts, the density of the tracts was low and the myocardium was still characterized by a very low FA and a high MD.

**Figure 5:**
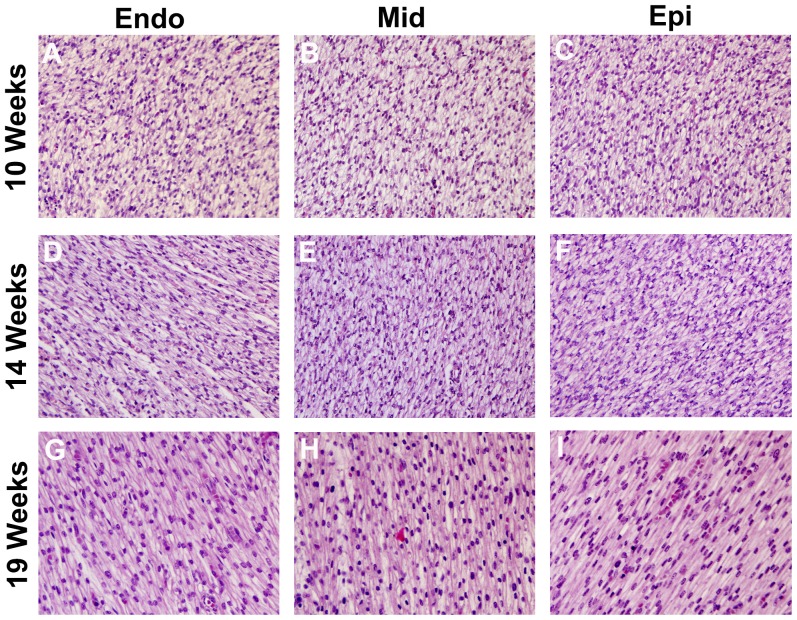
Histological sections of the lateral LV wall. The sections were cut tangential to the epicardial surface. (A–C) At 10 weeks the myocardium is isotropic with no clear structural pattern. (D–F) At 14 weeks structural anisotropy begins to evolve and distinct patterns of fiber orientation can be seen at different transmural depths. (G–I) By 19 weeks clear differences in fiber orientation can be resolved in all layers of the myocardium, and the alignment of the myofibers resembles that seen in the adult heart. However, no sign of a dense sheet structure is seen.

Histology of the lateral wall of the LV (Figure 5) confirmed the DTI findings. At 10 weeks, the fetal myocardium was highly isotropic with no discernable architectural pattern (Figure 5A–C). At 14 weeks, coherent patterns of myocyte alignment could be seen in some parts of the myocardium (Figure 5D–F). The characteristic transmural evolution of CM alignment was consistently seen at 19 weeks (Figure 5G–I).

## Discussion

The evaluation of myocardial architecture in the human fetal heart has been performed using histology and microscopy [13], [14]. These techniques, however, involve the destruction of the heart, are subject to tissue shearing/tearing and are two-dimensional. Here, for the first time we evaluate the microstructure of the human fetal heart with DTI. This allowed the hearts to be imaged in a completely intact state, 3D tracts of myofiber architecture to be resolved and measures of diffusion anisotropy to be quantified.

Our data suggest that fiber architecture in the human fetal heart develops in two distinct phases. At 10 weeks of gestation the myocardium is isotropic with no coordinated alignment of its CMs. Between 14 to 19 weeks of gestation the CMs align themselves into resolvable tracts that resemble those seen in the adult heart. However, in addition to FA, the λ_1_/λ_2_ and λ_1_/λ_3_ ratios remain low indicating that water diffusion has not yet become restricted by densely packed myofiber sheets. Sheet architecture, which is crucial to the function of a fully mature adult heart, thus develops relatively late in fetal life.

The evolution of fiber architecture in the fetal heart remains poorly understood and characterized. Studies in mice have shown that fetal CMs are spherical, and thus isotropic, until Carnegie Stage 18 of gestation [15]. At this time they begin to elongate, hypertrophy and form end-end connections supporting the generation of myofiber tracts. This process, however, is slow and is not yet complete at birth [15]. The results of our study suggest that a similarly slow process occurs in the human fetal heart. Myofiber anisotropy, and tracts resembling those seen in the adult heart, developed between 14–19 weeks of gestation, corresponding to the initiation of CM elongation in the human fetal heart. However, FA at 19 weeks remained extremely low, indicating that the CMs had only undergone a mild degree of elongation [11], [12].

Animal studies have shown that the cytoarchitecture of the heart is not mature at birth [15]. Further CM elongation and hypertrophy continue for at least the first month of post-natal life. Likewise, the fiber architecture of the neonatal pig heart has recently been shown to be highly plastic [16]. These findings are consistent with our observation that FA and the ratios of λ_1_/λ_2_ and λ_1_/λ_3_ in the P6 human heart were significantly lower than in the adult heart. The human heart thus likely also undergoes further structural maturation within the first months of life. This may have important implications for the timing of therapy in congenital heart disease.

Prior to the elongation of its constituent CMs the fetal heart undergoes four important processes: looping, trabeculation, septation and myocardial compaction [13]. These processes, however, are completed well before 10 weeks of gestation and are therefore unlikely to directly affect myocardial anisotropy [13]. Rather, it seems most likely that the process of CM elongation and polarization, which is most prominent in the latter stages of gestation [15], produces the 3D pattern of CMs seen in the adult heart. The signals responsible for this have not been fully elucidated, but likely involve the noncanonical Wnt planar cell polarity (PCP) pathway [17], [18], and possibly the ERK5 pathway [15].

The results of this study provide several intriguing insights into the structure-function relationship in the human fetal heart. Most intriguingly, the development of coherent myofiber tracts lagged behind the onset of cardiac contraction by 2–3 months [13]. It should be noted, however, that the extremely low impedance of the placenta provides a very low load against which the fetal heart contracts. Altering this load experimentally impacts CM orientation in the fetal heart [19], suggesting that mechanical forces may also play a role in CM alignment.

No evidence of a preexisting connective tissue scaffold with the ability to restrict water diffusion, and accordingly be resolvable with DTI, was seen in our study. This is consistent with prior observations that the deposition of the laminin network in the heart coincides with, and is a consequence of, CM elongation in the latter part of gestation [15]. This suggests that complex tissue scaffolds may not be a prerequisite for successful stem cell therapy in the heart. However, the developmental timeline of fiber tracts in the fetal heart suggests that a prolonged period (2–3 months) of preexisting cyclic contraction may be required to condition primitive CMs to mature, elongate and align.

The procurement of completely intact (non-dissected) human fetal hearts is extremely challenging and limited the number of hearts imaged in this study. Our findings, however, are in complete agreement with prior observations made by histology and microscopy [14]. This study is, to the best of our knowledge, the first application of diffusion tensor imaging and tractography in the human fetal heart, and provided valuable new insights into the transition of the heart from an isotropic tissue into a highly anisotropic organ. Moreover, while major technical hurdles remain, the performance of tractography of the human fetal heart *in-utero* is theoretically possible [20]. This could have profound implications for the diagnosis, treatment and prevention of congenital heart disease.
